# Availability, accessibility and delivery to patients of the 28 orphan medicines approved by the European Medicine Agency for hereditary metabolic diseases in the MetabERN network

**DOI:** 10.1186/s13023-019-1280-5

**Published:** 2020-01-06

**Authors:** Jean-Michel Heard, Charlotte Vrinten, Michael Schlander, Cinzia Maria Bellettato, Corine van Lingen, Maurizio Scarpa, the MetabERN collaboration group, Gert Matthijs, Marie-Cécile Nassogne, François-Guillaume Debray, Dominique Roland, Teodora Chamova, Viktor Kozich, Jesina Pavel, Martin Zenker, Martin Zenker, Christina Lampe, Anihb Martin Das, Julia Hennermann, Stefan Kölker, Natalie Weinhold, Klaus Mohnike, Sarah Gruenert, Allan Meldgaard Lund, Montserrat Morales-Conejo, Mireia del Toro-Riera, Luis Aldámiz-Echevarría, Maria-Teresa Garcia-Silva, Manuel Schiff, Laurent Gouya, Pascale de Lonlay, Nadia Belmatoug, Dominique P. Germain, Aline Cano, Dries Dobbelaere, Simon Jones, Charlotte Dawson, Patrick Deegan, Saikat Santra, Suresh Vijay, Danijela Petkovic Ramadza, Ivo Barić, Tamara Žigman, György Pflieger, Katalin Szakszon, Rita Kaposta, Serena Gasperini, Alberto Burlina, Giancarlo Parenti, Pietro Strisciuglio, Giovanni Ceccarini, Antonio Federico, Alessandro Simonati, Birute Tumiene, Hidde Huidekoper, Francian van Spronsen, Annet Bosch, Maria-Estela Rubio-Gozalbo, Gepke Visser, Trine Tangeraas, Aasne Aarsand, Beata Kieć-Wilk, Ana-Maria Simões Mendes Gaspar, Dulce Quelhas, Elisa Leao-Teles, Olga Azevedo, Esmeralda-Maria Ferreira Rodriges Silva, Luísa-Maria de Abreu Freire Diogo Matos, Esmeralda Martins, Svetlana Lajic, Niklas Darin, Urh Groselj, Mojca-Zerjav Tansek

**Affiliations:** 1 MetabERN, Regional Coordinating Center for Rare Diseases, Udine University Hospital, Piazzale Santa Maria della Misericordia, 15, 33100 Udine, Italy; 2 Imperial College Business School, London, UK; 3 Division of Health Economics, German Cancer Research Center, Heidelberg, Germany

**Keywords:** Orphan medicinal product, Access to treatment., European Reference Network., Hereditary Metabolic Diseases., Inborn errors of metabolism.

## Abstract

**Background:**

The European Medicine Agency granted marketing approval to 164 orphan medicinal products for rare diseases, among which 28 products intended for the treatment of hereditary metabolic diseases. Taking advantage of its privileged connection with 69 healthcare centres of excellence in this field, MetabERN, the European Reference Network for hereditary metabolic diseases, performed a survey asking health care providers from 18 European countries whether these products are available on the market, reimbursed and therefore accessible for prescription, and actually delivered in their centre.

**Results:**

Responses received from 52 centres (75%) concerned the design of treatment plans, the access to marketed products, and the barriers to delivery. Treatment options are always discussed with patients, who are often involved in their treatment plan. Most products (26/28) are available in most countries (15/18). Among the 15 broadly accessible products (88.5% of the centres), 9 are delivered to most patients (mean 70.1%), and the others to only few (16.5%). Among the 10 less accessible products (40.2% of the centres), 6 are delivered to many patients (66.7%), and 4 are rarely used (6.3%). Information was missing for 3 products. Delay between prescription and delivery is on average one month. Beside the lack of availability or accessibility, the most frequent reasons for not prescribing a treatment are patients’ clinical status, characteristic, and personal choice.

**Conclusions:**

Data collected from health care providers in the MetabERN network indicate that two-third of the orphan medicines approved by EMA for the treatment of hereditary metabolic diseases are accessible to treating patients, although often less than one-half of the patients with the relevant conditions actually received the approved product to treat their disease. Thus, in spite of the remarkable achievement of many products, patients concerned by EMA-approved orphan medicinal products have persistent unmet needs, which deserve consideration. The enormous investments made by the companies to develop products, and the high financial burden for the Member States to purchase these products emphasize the importance of a scrupulous appreciation of treatment value involving all stakeholders at early stage of development, before marketing authorization, and during follow up.

## Background

Diseases affecting less than 200,000 people in the United States (US), or less than one person per 2000 inhabitants in the European Union (EU), are considered « rare diseases ». There are more than 7000 rare diseases affecting between 27 to 36 million people in the EU [1]. Appropriate treatments are available for less than 5% of rare diseases. Due to the high number and low prevalence of these conditions, the research efforts to find a therapeutic strategy and the development of potential products for marketing are limited to a small proportion of the best candidate diseases. To deal with this problem, public intervention has established legal incentives to create an attractive environment for the pharmaceutical industry to develop and market drugs for rare diseases, and to assure patients with rare diseases a remedy for their illnesses. These incentives are the raison d’être of the orphan drug regulations implemented in the US, the EU and Japan. These regulations were reviewed previously [2–6]*.*

The European orphan medicinal products (OMPs) regulation entered into force in January 2000 [7]. It establishes the criteria for OMP designation, created a new body within the European Medicines Agency (EMA), called the Committee for Orphan Medicinal Products (COMP), and defined a set of incentives accruing to designated OMPs in the EU. The law refers to OMPs as “*any substance or combination of substances which may be administered to human beings with a view to making a medical diagnosis or for treating or preventing a disease*” (medical devices and nutrition supplements are not covered). To benefit incentives the sponsor must establish: i) that the product addresses a life threatening or chronically debilitating condition affecting no more than five in 10 thousand persons; ii) that without incentives it is unlikely that the marketing of the product would generate sufficient return to justify the necessary investments; iii) that either no satisfactory method of diagnosis or treatment of the condition exists, or the product will be of significant benefit for the patients.

Between 2000 and 2018, the EMA has granted 2121 orphan designations, and 164 OMPs for 124 conditions have obtained marketing authorization across the EU [8, 9]. The European Commission regularly publishes inventories of the incentives to support research, development and availability of OMPs in the Member States. The 2015 inventory emphasizes the steady increase in the number of designation requests over the years [10]. The economic and societal impacts of the orphan medicine regulation, as well as the availability of, and accessibility to approved OMPs in the EU have been examined [3, 11–13]. These analyses concluded that the Orphan Medicines Regulation led to significant benefits for patients and has a positive economic impact on small, medium and larger pharmaceutical companies. They also pointed out that the medicines may not be effective for all patients with the disease, and that not all patients have effective access to the treatments, with variations between European countries. Data used in these various studies were extracted from information on approval and reimbursement available in the publications of European and national public bodies. With regard to their privileged access to medical teams specialized in the management of rare diseases throughout the EU, the European Reference Networks (ERNs) are well suited to collect information directly from the health care providers (HCPs). ERNs can therefore bring complementary information on the availability of OMPs (whether marketing is authorized in the ERN participating countries), the accessibility to OMPs (whether the product is reimbursed by national health systems), and to which extent it is actually used to treating patients (whether the product is prescribed and delivered to a significant proportion of patients with the relevant condition).

Twenty-four ERNs were launched as a result of the adoption of Directive 2011/14/EU on patient’s rights in cross-border healthcare [14]. They became operational in 2017, and represent more than 900 Centres of Excellence in various fields of diagnosis, management and care of rare disease patients. They are located in 313 hospitals in 25 Member States, plus Norway. MetabERN is the ERN for Hereditary Metabolic Diseases (HMDs). It consists in 69 centres in 17 Member States, plus Norway, mostly belonging to University hospitals, in which 1671 professional follow more than 40,000 patients with HMDs. HMDs represent 11% of the OMP designations, and the 28 OMPs marketed for the treatment of HMDs represent 17% of all EMA-approved OMPs. MetabERN made use of its privileged access to the HMD community throughout the EU to enquire its members about the prescription of these 28 OMPs. We report here the results of a survey that was sent to the 69 centres of the MetabERN network for that purpose in July 2018.

## Results

A questionnaire comprised of 31 questions (4 general questions, 25 multiple choices questions, 2 open questions, shown in supplementary material, Additional file 1: Table S1) was addressed to the 69 MetabERN Centres of Excellence. HCPs were asked about interactions between physicians and patients for the design of a treatment plan, and for each of the 28 EMA-approved OMP, whether they are marketed and accessible in the country, how many patients receive this treatment, and what are the barriers limiting delivery to patients, including with regard to possible delays in making the product available and accessible to prescription. Responses were received from 52 centres (75% of the MetabERN centres), including at least one responding centre for each of the 18 countries participating in MetabERN (supplementary material, Additional file 1: Table S2). Some centres returned several responses. There was no inconsistency within multiple responses from the same center. A total of 65 responses were analyzed, among which 50 questionnaires were fully completed. At least one complete data set was returned from 16 countries (exceptions are NO and SE).

### Interactions between physicians and patients for the design of a treatment plan

We received 65 responses to the questions related to the interactions between physicians and patients (questions 13–17 in Additional file 1: Table S1). All respondents declared that a multidisciplinary team is in charge of the definition of the treatment plan in their centre. Treatments are prescribed to both adults and pediatric populations (*n* = 63, 97%). A specific adult unit is available in many centres (*n* = 52, 80%), although many pediatricians can prescribe treatment to adults (*n* = 43, 66%), with a transition program often in place (*n* = 34, 52%).

All physicians discuss different therapeutic options with patients before treatment is proposed (*n* = 65, 100%). It is common that HCPs spent 20 to 40 min (*n* = 42, 64%) or more (*n* = 20, 31%) discussing treatment options with their patients. All respondents estimate that they have enough time to discuss this issue properly. Information on their disease and treatment options is provided to patients during face-to-face meetings (n = 65, 100%), although leaflets (*n* = 57, 88%), or referral to a patient’s groups (*n* = 53, 81%) are also commonly used.

Patients are always involved in the design of their treatment plan (n = 65, 100%). In the event where a treatment has to be stopped because of the progression of the disease, this decision is most of the time discussed with the patients, or their family members (*n* = 48, 74%).

### Access to OMPs and prescription

The questionnaire included the list of the 28 EMA-approved OMPs for HMDs (supplementary material, Additional file 1: Table S3). We received 54 responses to the questions related to the availability and delivery to patients of these medicines at the national and local levels (questions 18–25 in Additional file 1: Table S1).

In a previous survey performed in 2017, in the year MetabERN was created, a questionnaire was sent to HCPs in each participating centre with the aim to get an estimate of the number of patients per HMD condition they followed. With respect to the current study, this information provided an indication of the numbers of patients registered in the active dossiers of the MetabERN centres who have a condition corresponding to the therapeutic indications of each of the 28 considered EMA-approved OMPs. This estimated number of followed patients was compared with the estimated number of patients receiving a given OMP, as declared by the respondents to the present survey, thus providing a gross estimation of the proportion of treated patients for each considered condition.

Table 1 shows accessibility to EMA-approved OMPs in all the responding centres taken together. The results are the percentages of respondents (*n* = 54) declaring that a given OMP is accessible in their country of residence (National) and/or in the centre where they practice (Responding centre). Data indicate that 15 OMPs (53%) are accessible in a large majority of the responding centres (88.5%), 7 OMPs (25%) are accessible in about one-half of the centres (52%), and 3 OMPs (11%) are accessible in only few centres (15%). The estimated numbers of patients with conditions corresponding to the therapeutic indications of each OMPs who are followed in the responding centres is shown in Table 1, as well as the estimated numbers of patients receiving the treatment in these centres. It is noticeable that only five products (Nitisinone, Chenodeoxycholic acid, Alglucosidase alpha, Idursulfase and Galsulfase) seem to be prescribed to almost the entire population of followed patients, whereas the other medicines are seemingly rarely given to more than one-half of the followed-up patients, and some appear rarely prescribed, or not prescribed at all.

**Table 1:** Accessibility and prescription of EMA-approved OMPs for HMDs in the MetabERN centres

Active substance	Trade name	Accessibility^a^		Indication	Number of patients in MetabERN ^b^		
		National	Centre		Total (in 2017)	Treated (in 2018)	Estimated proportion
Carglumic acid	Carbaglu Ucedane	83%	81%	Organic aciduria	530	87	< 1/3
Glycerol Phenylbutyrate	Ravicti	63%	59%	Urea cycle	785	115	< 1/3
Sodium Phenylbutyrate	Buphenyl Ammonaps	85%	82%			379	> 1/3
Sapropterin	Kuvan	78%	76%	PKU	4539	365	< 1/3
Nitisinone	Nitisinone Nytir Orfadin	85%	80%	Tyrosinemia	208	257	> 1/3
Cholic acid	Cholbam Orphacol	52%	50%	Bile acid synthesis type 1	–	11	–
Chenodeoxycholic acid	Chenodeoxycholic acid Leadiant	65%	57%	Cerebrotendinous Xanthomtosis	25	47	> 1/3
Alipogene tiparvovec	Glybera	17%	15%	Lipoprotein lipase deficiency	30	0	< 1/3
Cysteamine hydrochl.oride	Cystadrops	52%	43%	Cystinosis	87	41	> 1/3
Cysteamine bitartrate	Cystagon	61%	59%			73	> 1/3
Migalastat	Galafold	76%	70%	Fabry disease	1361	76	< 1/3
Agalsidase alpha	Replagal	87%	87%			521	> 1/3
Agalsidase beta	Fabrazyme	89%	89%			376	< 1/3
Eliglustat	Cerdelga	81%	72%	Gaucher disease	641	125	< 1/3
Imiglucerase	Cerezyme	89%	83%			273	> 1/3
Velaglucerase	VPRIV	87%	80%			142	< 1/3
Miglustat	Zavesca	94%	93%	NPC	188	99	> 1/3
Sebelipase alpha	Kanuma	63%	52%	Wolman disease	58	39	> 1/3
Cerliponase	Brineura	43%	35%	CNL2	17	10	> 1/3
Alglucosidase alpha	Myozyme	87%	81%	Pompe disease	268	305	> 1/3
Laronidase	Aldurazyme	87%	81%	MPS I	302	153	> 1/3
Idursulfase	Elaprase	89%	83%	MPS II	193	146	> 1/3
Elosulfase	Vimizin	67%	57%	MPS IVa	198	112	> 1/3
Galsulfase	Naglazyme	76%	74%	MPS VI	117	107	> 1/3
ADA CD34+ cells	Strimvelis	18%	9%	SCID, ADA deficiency	9	0	< 1/3
Idebenone	Mnesis, Raxone	80%	70%	Leber Hereditary Optic Neuropathy (LHON)	–	85	–
Afamelanotide	Scenesse	26%	15%	Erythropoietic. Protoporphyria (EPP)	281	128	> 1/3
Asfotase alpha	Strensiq	46%	30%	Hypophosphatasia.	–	5	–

^a^ Accessibility is indicated as the percentage of respondents declaring that the considered product is accessible for prescription in the country (National), or in the centre (Centre) where they practice

^b^ At the time the MetabERN network was launched (2017), the participating centres provided information about the numbers of patients with each individual HMD they followed. The numbers of patients followed in the centres that responded to the current survey was established accordingly, giving a gross estimation of the number of patients who were potential candidates to receiving the indicated treatment in the responding centres in 2017 (this information is missing for bile acid synthesis type 1 defects, hypophosphatasia, and Leber hereditary optic neuropathy). The number of patients receiving the listed products in these centres in 2018 was declared by HCPs in response to the current survey. Data were received from 15 among the 18 participating countries, with the exceptions of NO and SE. For 3 products (Nitisinone, Chenodeoxycholic acid and Alglucosidase alpha) the declared number of patients treated in 2018 is higher than the number of patients followed in the centres in 2017. A gross estimation of the proportion of treated patients is indicated as being either lower, or higher than one-third of the total number of patients with the relevant condition

Table 2 shows the results for individual countries. They indicate that at least 26 of the 28 EMA-approved OMPs are available in 15 of the 18 countries participating in MetabERN. The products most often unavailable are gene therapy medicines (Alipogene tiparvovec and ADA CD34+ cells), Afamelanotide, Asfotase alpha, or Cholic acid. Whereas only 10 EMA-approved OMPs are available in BG, most are available in the other EU13 countries participating in MetabERN (CZ, HR, PL, and SL). Open boxes in Table 2 indicate that although the drug is available, respondents were unable to indicate whether patients actually receive the treatment in their country, such as information about the accessibility of the product in the country is missing. We could not specify whether the absence of answer is because HCPs do not themselves follow patient with the condition, whereas the product can be prescribed by other physicians, or because the product is not accessible for prescription in their centre. Commonalities of prescription between countries are visible. There are drugs prescribed to a high proportion of patients almost everywhere, like enzyme replacement therapies for certain lysosomal storage diseases, and to a lesser extend Nitisinone for tyrosinemia. On the other end, differences between countries exist for certain products. Cerliponase for CNL2 is prescribed in FR, although it was apparently rarely used in 2018 in MetabERN centres located in other countries. Cysteamine bitartrate for cystinosis is given to a majority of patients in many countries (CZ, DE, DK, ES, PL, PT, SL), but not in others (FR, NL, GB). The prescription of Afamelanotide for erythropoietic protoporphyria was reported to be used only at the Erasmus Medical Center in the NL.

**Table 2:**
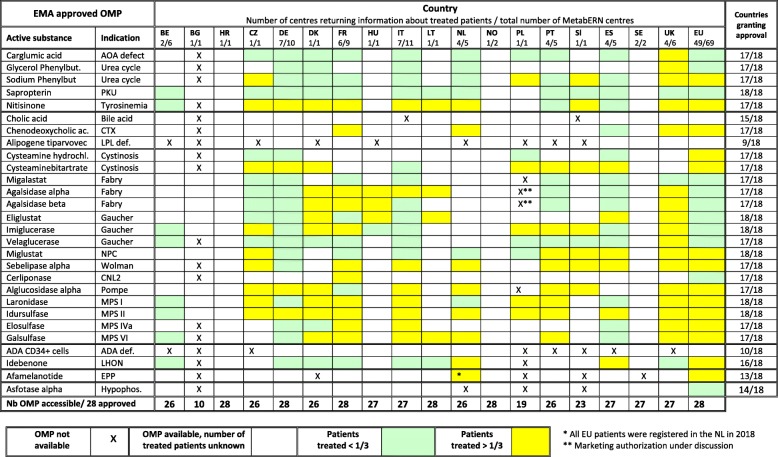
Accessibility and prescription of EMA-approved OMPs for HMDs in the 18 individual MetabERN participating countries

* All EU patients were registered in the NL in 2018

** Marketing authorization under discussion

According to these results, OMPs can be attributed to arbitrary categories depending on whether they are accessible in more or less than two-third of the MetabERN centres, and whether they are delivered to more or less than one-third of the patients with the condition for which they are indicated (Table 3). Ten OMPs are broadly accessible and delivered to a significant proportion of patients with the target condition (Table 3, first column). Five OMPs are broadly accessible, though delivered to a small proportion of patients (Table 3, second column). Seven OMPs are not broadly accessible, although frequently delivered to patients when they are (Table 3, third column). Three products are not accessible in several countries and delivered to only few or no patients in the countries where they are accessible (Table 3, fourth column). Data on the total number of patients followed in the centres are missing for 3 conditions (bile acid synthesis type 1 defects, hypophosphatasia, and Leber hereditary optic neuropathy), thus impairing estimation of the proportion of treated patients.

**Table 3:** Categories of EMA-approved OMPs for HMDs according to accessibility and delivery to patients in the MetabERN centres

OMP accessible in > 2/3 of the centres (mean = 88.5%)		OMP accessible in < 2/3 of the centres (mean = 40.2%)	
Delivered to > 1/3 of the patients (mean = 70.1%, range 38–100%)	Delivered to < 1/3 of the patients (mean = 15.8% range 7–23%)	Delivered to > 1/3 of the patients (mean = 66.3% range 45–100%)	Delivered to < 1/3 of the patients (mean = 6.0% range 0–18%)
Sodium Phenylbutyrate	Carglumic acid	Chenodeoxycholic acid •	Glycerol phenylbutyrate
Nitisinone •	Sapropterin	Cysteamine hydrochloride	
Miglustat	Migalastat	Cysteamine bitartrate	Alipogene tiparvovec
Agalsidase alpha	Eliglustat	Cerliponase	ADA CD34 cells
Agalsidase beta	Velaglucerase	Elosulfase	
Imiglucerase		Sebelipase	
Alglucosidase alpha *		Afamelanotide	
Laronidase			
Idursulfase •			
Galsulfase •			

Information about the number of patients followed in the centres is missing for Cholic acid (accessible in more than 2/3 of the centres), Idebenone (accessible in less than 2/3 of the centres) and Asfotase alpha (accessible in less than 2/3 of the centres). • indicates products prescribed to almost all the patients with the considered condition

### Barriers to delivery

We received 54 responses to the questions related to possible barriers to EMA-approved OMPs delivery to patients (questions 26–31 in Additional file 1: Table S1). Most respondents estimated that barriers actually restrict delivery to patients (*n* = 45, 83%).

An obvious barrier to the prescription of EMA and nationally approved products is the lack of inclusion on the national list of reimbursable medicines. This is considered as a barrier by about one-third of the respondents (*n* = 16, 30%). More respondents estimate that delay for inclusion on the list of reimbursed products is an important barrier to accessibility (*n* = 22, 41%). Since delay most often corresponds to pricing negotiation, it is assumable that high price causes longer delay, although this issue was not addressed by the survey. With regard to the delay due to negotiation and its possible negative outcome, many HCPs consider that budget constraints are a barrier to prescription (*n* = 21, 39%).

When inclusion on the list of reimbursed products is effective, delay between prescription and delivery to the patient is usually less than 3 months, and often less than one month, in most countries (Table 4). Delay between prescription and delivery to patients is therefore not a serious barrier in many cases, although exceptions exist for some products in certain countries.

**Table 4:**
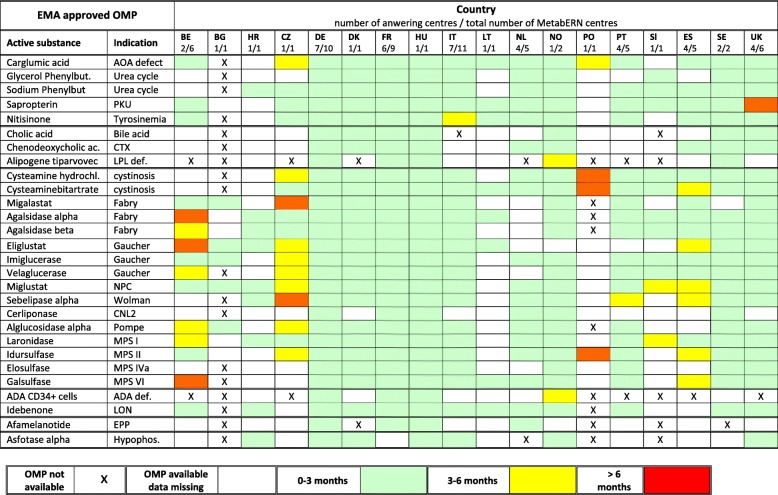
Delay to initiate treatment with EMA-approved OMPs for HMDs following prescription in the MetabERN participating countries

Important barriers to prescription are related to the expected benefit to the patient. Respondents considered that the most frequent reasons for not prescribing EMA-approved OMPs are patient’s clinical status (*n* = 34, 63%), patient’s personal choice (*n* = 26, 48%), and individual characteristics of the patient (*n* = 22, 40%), which may be worsened by late referral to a specialized centre (*n* = 21, 39%). Taken together, these factors limit the therapeutic benefit that can realistically be expected from the treatment.

The questionnaire ended with two open questions inviting respondents to suggest means to overcome the challenges surrounding access to treatment and to improve delivery of EMA-approved OMPs to patients. Responses (*n* = 29) suggested two directions for improvement. A first direction concerned economical issues (*n* = 19). Respondents considered that a more accurate regulation of pricing and reimbursement rules would be beneficial. Ideally, it could be shared between all EU countries and would ensure immediate reimbursement of treatment by national health care payers. A second direction focused on the clinical value of OMPs (*n* = 16). Respondents considered that the development of treatments that are better adapted to patient’s clinical conditions and needs is desirable. They estimated that this would require improved knowledge, understanding and consideration of rare disease conditions by public and private decision-makers. They also emphasized on the value of European guidelines thoroughly specifying the clinical indications of OMPs, the criteria to be retained for their prescription, and the modalities of patient’s follow-up.

## Discussion

ERNs are unique instruments to collect information on health care practices in the field of rare diseases directly from the end-users, i.e., the HCPs and the patients. We took advantage of the MetabERN network to document the availability, accessibility and delivery to patients of 28 marketed OMPs for HMDs. Data were directly collected from HCPs in 52 healthcare centres specialized in the management of these diseases throughout 18 countries of the EU.

Information collected in the field through surveys is intrinsically subjective, as it reflects the personal perception and experience of the respondents. Thus, it cannot replace information from official statistics of medicine consumption, official medical agency reports, reimbursement records of national public healthcare payers, or financial balance sheets of pharmaceutical companies. Information collected in the field may nevertheless be useful to complement the pictures drawn from objective indicators of medical activity with the views of professionals confronted with the daily practice of care. With a similar approach, Eurordis repeatedly addressed surveys to patients and families on access to treatments [15, 16].

The information collected through the current survey has limitation. It is not representative of the situation in the EU as a whole, since centres participating in MetabERN are present in only 17 of the 28 member states plus Norway. Countries like AT, FI, GR, IE, as well as several of the EU13 countries, are not represented in the network. Thus, although most of the patients diagnosed with HMDs in the represented countries are referred to one of the specialized MetabERN centres, these centres cover only about 80% of the EU population, with unbalanced representation of the EU13 and EU15 parts of the EU. MetabERN being a network focused on HMDs, the information collected is relevant to this field only, and cannot be extrapolated to other rare diseases. As HMDs are polymorphic, patients with HMDs may be referred to centres not specialized in HMDs, which did not participate to the survey. The information collected on certain diseases was therefore not exhaustive. This likely includes diseases in which neurological (CNL2), immunological (SCID ADA), hematological (erythropoietic protoporphyria), ophthalmological (LHON) or skeletal (hypophosphatasia) manifestations predominate. The collected information must also be taken with caution because of possible bias. Multiple answers from some centres and incomplete answers from others may affect the global picture. Answers to the questionnaire are often an approximation and are always affected by HCPs’ personal experience and expertise, the specialization of the institution, and the national modalities for referring patients. Missing information on the declared numbers of treated patients is relatively high, as shown by the open boxes in Table 2. Most of the time, respondents do not have the information because the disease is outside of their personal expertise. However, when several respondents with different areas of expertise, working in different centers in the same country are all unable to specify whether patients are treated, open boxes in Table 2 likely suggest that the OMP is not accessible, or rarely delivered to patients in this country.

The survey shows that marketing approval by EMA was most often followed by approval by national authorities. This concerns 26 out of the 28 OMPs considered in this study, exceptions being the two gene therapy products, Alipogene tiparvovec (withdrawn from the European market in 2017) and ADA CD34+ cells. Most EMA-approved OMPs are thus marketed in 15 of the 18 countries of the MetabERN network, with the noticeable exceptions of BG, and to a much lesser extent of PL and SL. With these exceptions in mind, it can be considered that EMA-approved OMPs for HMDs are broadly available in the MetabERN network. However, the survey provides evidence that availability on the market does not guarantee access and delivery to patients.

Lack of reimbursement is an important barrier to the delivery of OMPs. Variation of reimbursement rules between European countries has been previously documented (12, 13, 16). It is interesting to compare our results with the analysis of Malinovsky et al. [12], who examined the reimbursement of 16 of the EMA-approved OMPs for HMDs that we investigated in 10 of the countries where we collected information. Although data are often consistent, we noticed 21 cases in which information collected from health authorities by Malinovsky et al. indicated a lack of reimbursement in 2016, whereas HCPs said that they prescribed the product in 2018. Whereas rules may have changed in the meantime, discrepancies may also appear because of the multiplicity and complexity of reimbursement rules in Europe, which consist not only in the registration on the national list of reimbursable medicines, but also in compassionate and off-label uses, temporary authorizations, or other schemes. The existence of such procedures was acknowledged by almost all respondents to the survey (*n* = 49/54). This observation emphasizes the value of confronting information collected from official bodies with those coming from HCPs. Our results are consistent with the survey of patients performed by Eurordis in 2010 with the collaboration of ten National Alliances of patient’s organizations [15], which emphasized the privileged status of HMDs with regards to the availability of and accessibility to OMPs. Our survey brings the additional point of view from HCP’s experience, thus providing insights into how available and accessible treatments for HMDs are actually delivered to patients.

The survey brings light on the effective delivery to patients of 25 EMA-approved OMPs for HMDs, whereas information is missing for 3 products (Table 3). Fifteen products are accessible to prescription in a large proportion of the MetabERN centres (88.5% of the centres), among which 10 products are delivered to a high proportion of patients, and 5 to only few. Ten other products are accessible in less than two-third of the MetabERN centres (40.2% of the centres). In spite of this limitation, 7 are delivered to a significant proportion of patients, suggesting that HCPs considered clinical benefits sufficient to systematically propose this treatment to their patients, when accessible. Delivery to more patients is presumably limited by the lack of reimbursement in several countries. Three products are hardly accessible and rarely prescribed.

As a whole, the 25 EMA-approved OMPs for which relevant information could be collected comprise products delivered to the entire patient population (*n* = 5), products delivered to one-half of the patient population (*n* = 12), and products rarely delivered to patients (*n* = 8). The five products prescribed to almost all patients when accessible (Nitisinone, Chenodeoxycholic acid, Alglucosidase alpha, Idursulfase and Galsulfase) are clearly unavoidable. The twelve products delivered to about one-half of the patient population (mean: 54.1%, range: 38–70%) are obviously of high value, although prescription may be limited by an existing treatment of reference, as hematopoietic stem cell transplantation for MPS I in the case of Laronidase, or because the treatment is active on acute phases, disease complications, or as a complementary therapy. Patients with the relevant conditions may also be referred to centres not participating to MetabERN. However, patient’s clinical status seems to be the most frequent limit to the prescription of these treatments. This presumably includes not addressable neurological manifestations and/or advanced clinical degradation. Barriers may also come from treatment-associated side effects or logistical constraints, especially for patients with particular characteristics or marked risk avoidance. Noticeably, Agalsidase alpha and Agalsidase beta, which are two very similar products in this group, are delivered to almost all patients with Fabry disease if considered together. The eight OMPs that are delivered to a small proportion of the patient population (mean = 7.1%, range 0–23%) are either products duplicating an existing drug (“me too”, i.e. Velaglucerase duplicating Imiglucerase for the treatment of Gaucher disease), or treatment for which clinical benefit is currently considered marginal or insufficient with respect to treatment risk, treatment burden, and/or cost.

Data collected from HCPs in the MetabERN network indicate that two-third of the orphan medicines approved by EMA for the treatment of HMDs are efficiently delivered to patients, although in many cases, slightly more than one-half of the patients with the relevant conditions actually received the approved product to treat their disease. As several of the considered OMPs dramatically improve the quality of life of patients, incentives to develop these products can be seen as a major contribution of the OMP regulation to rare disease clinical management. However, the present study also points out persistent unmet needs of patients concerned by EMA-approved OMPs. With respect to the enormous investments consented by the companies to develop these products for marketing, and to the high financial burden for the Member States to purchase them, persistent unmet needs cannot be ignored. They are rarely due to treatment unavailability, slightly more often to restricted accessibility in certain countries. They are actually more often related to the insufficient benefit brought by the treatments, at least for certain individual patient’s conditions.

## Conclusion

It was not the scope of this study to examine the impact of treatment costs, which was debated elsewhere [17–22], but rather to bring some indications on benefits for patient populations that are relevant to the assessment of treatment value [23]. More accurate assessment of patient’s population benefit would require measuring markers of disease natural history and patient’s quality of life in treated patient cohorts and analysis of the results by all stakeholders, including HCPs, patients and families. Such methodological approach is needed to ensuring appropriate assessment of marketed treatment value and adapted decision on reimbursement. It is also desirable that end-users and public policy makers are involved at early steps of product development, in order to estimate the potential and/or expected value of the candidate treatments that are selected for development and future marketing. This might reduce the risk of developing and marketing products that do not adequately meet patient’s needs, and might optimize priority investments for OMPs.

## Methods

The MetabERN network has been described previously [24], and the list of the MetabERN centres that responded to the survey is available as supplementary material (Additional file 1: Table S4).

The MetabERN coordination team designed the survey, which was reviewed by HCP’s and patient’s representatives. The survey was performed in July 2018, asking the 69 MetabERN centres to complete questionnaires on the online SurveyMonkey platform (supplementary material, Additional file 1: Table S1). The questionnaire was in English. Sixty-five individual answers were received (7 out of the 72 filled questionnaires returned on the plateform were duplicated) from 52 centres (41 centres returned one questionnaire, 12 centres returned 2 questionnaires, one centre returned 3 questionnaires, Additional file 1: Table S4). Fifty questionnaires were fully completed.

Sixty-five answers from the 52 responding centres concerned the development of treatment plan and the interactions between physicians and patients. There was no inconsistency in the various answers received from the same centre. Since there are several questions concerning the individual practice of HCPs, the results are given according to the number of responses received.

Fifty-four answers from 49 centres in 16 countries provided information on the number of treated patients and the barriers to delivery. Each response was examined individually. When several answers were received from the same centre (*n* = 4), the highest declared numbers of patients were considered. This information was missing for NO and SE. The numbers of treated patients declared by the responding centres was compared with the numbers of patients with the considered condition followed by the same centres in 2017, when the MetabERN network was launched. As more patients have been recruited during the meantime, in 3 cases (Nitisinone, Chenodeoxycholic acid and Alglucosidase alpha), there were more patients treated in 2018, than patients declared with the relevant conditions in 2017. This situation was interpreted as an indication that the entire patient population received the treatment. There was no inconsistency in the various answers received from the same centre regarding the appreciation of the barriers to delivery.

## Supplementary information

**Additional file 1: Table S1**. Questionnaire addressed to MetabERN centres. **Table S2.** Responding centres. **Table S3.** List of the 28 EMA-approved OMPs for HMDs. **Table S4.** List of MetabERN centres and number of questionnaires completed

## Data Availability

The datasets used and/or analyzed during the current study are available from the corresponding author on reasonable request.

## Abbreviations

ADA: Adenosine deaminase
AOA: Aminoacids and organic acids related disorders
AT: Austria
BE: Belgium
BG: Bulgaria
CDG: Congenital disorders of glycosylation and disorders of intracellular trafficking
C-FAO: Carbohydrate, fatty acid oxidation and ketone bodies disorders
CNL: Ceroidneurolipofuscinosis
COMP: Committee for Orphan Medicinal Products
CTX: Cerebrotendinous xanthomatosis
CZ: Czech Republic
DE: Germany
DK: Denmark
EMA: European Medicines Agency
EPP: Erythropoietic protoporphyria
ERN: European Reference Network
ES: Spain
EU: European Union
FI: Finland
FR: France
GR: Greece
HCP: Health Care Provider
HMD: Hereditary Metabolic Disease
HR: Croatia
HU: Hungary
IE: Ireland
IT: Italy
LHON: Leber hereditary ophatalmic neuropathy
LPL: Lipoprotein lipase
LSD: Lysosomal Storage Disease
LT: Lithuania
MPS: Mucopolysaccharoridosis
MS: Member State
NL: Netherlands
NO: Norway
NOMS: Disorders of Neuromodulators and Small Molecules
NPC: Nieman-Pick disease
OMP: Orphan medicinal product
PD: Peroxisomal and lipid related disorders
PKU: Phenylketonuria
PKU: Phenylketonuria
PL: Poland
PM-MD: Disorders of pyruvate metabolism, Krebs cycle defects, mitochondrial oxidative phosphorylation disorders, disorders of thiamine transport and metabolism
PT: Portugal
RO: Romania
SE: Sweden
SL: Slovenia
UK: United Kingdom
