# Supplementary material for: Availability, accessibility and delivery to patients of the 28 orphan medicines approved by the European Medicine Agency for hereditary metabolic diseases in the MetabERN network
Source: Orphanet J Rare Dis. 2020 Jan 6;15:3. doi: 10.1186/s13023-019-1280-5 (PMC6945588; doi:10.1186/s13023-019-1280-5)
Supplement: Supplementary file 1 — Additional file 1: Table S1. Questionnaire addressed to MetabERN centres. Table S2. Responding centres. Table S3. List of the 28 EMA-approved OMPs for HMDs. Table S4. List of MetabERN centres and number of questionnaires completed [file 13023_2019_1280_MOESM1_ESM.docx]

**Supplementary material**

**Table S1 : Questionnaire addressed to MetabERN centres**

| **Q** | **GENERAL DATA** |
| --- | --- |
| 1 | Do you agree to the above terms? |
| 2 | Profession |
| 3 | Organization Name |
| 3 | Country of Residence |
| 4 | Area of expertise from list of diseases* |
|  | **DEVELOPMENT OF TREATMENT PLAN** |
| 5 | Is a multidisciplinary team involved in the treatment plan of your patient? |
| 6 | Do you discuss different treatment options (eg. pharmacological or non-pharmacological) with your patients before selecting the treatment? |
| 7 | Are patients involved in the larger development of their treatment plan? |
| 8 | Can your Centre prescribe treatment to both adult and paediatric populations? |
| 9 | Is there an Adult Unit in your Centre |
| 10 | Can you as a paediatrician prescribe treatment to adults? |
| 11 | If yes, do you have a transition program in place? |
| 12 | If no, who is in charge of prescribing treatment to adults? |
|  | **INTERACTIONS DOCTORS-PATIENTS** |
| 13 | How do you convey information on treatment to your patients? (please tick as it applies):   - Workshops - Leaflets/Written information - Face-to-face meeting - Referral to patient groups - Other (please specify) |
| 14 | How much time do you currently spend discussing treatment options with your patients? (multiple choice answer)   - 0-20 minutes - 20-40 minutes - More than 40 minutes |
| 15 | Do you feel the time spent discussing treatment options with your patients is enough? (Slider: Not at all – Somewhat – A lot) |
| 16 | Have you ever decided to stop the treatment of a patient because of the progression of the disease? |
| 17 | If yes, did you discuss this decision with patients or their family members? |
|  | **ACCESS TO PHARMACOLOGICAL TREATMENT** |
| 18 | For the following EMA-approved treatments, please indicate which are available for prescription in your COUNTRY. Please select as many as apply** |
| 19 | Following EMA approval, are there any national and regional bodies in your country in charge of establishing prices, reimbursement, special rules for drug use and access to the market? |
| 20 | Are you aware of orphan drugs approved by the FDA, which are not currently approved by the EMA, and vice-versa? |
| 21 | For the following EMA-approved treatments, please indicate which are available for prescription in your TREATMENT FACILITY. Please select as many as apply** |
| 22 | For the treatments available in your facility, could you please indicate how many patients have access to them? Please express a number for each of the fields. If data are unavailable please write 0 or not available ** |
| 23 | For those treatments available, how long does it take on average to patients to get access to therapy? Please respond to each field (multiple choice answer)**   - 0-1 month - 1-3 Months - 3-6 Months - 6-12 Months - 1-2 Years - More than 2 years |
| 24 | For the following treatments, are there any clinical criteria based on which you decide the INCLUSION of patients in treatment schemes? Please answer to as many as apply** |
| 25 | For the following treatments, are there any clinical criteria based on which you decide the EXCLUSION of patients from access to treatment? Please answer to as many as apply ** |
| 26 | For those pharmacological treatments that are available in your facility, are there any barriers to prescription? (please tick as it applies)   - Patients' clinical status - Patients' characteristics - Patients' choice - Budget constraints - There are no barriers - Other, please explain |
| 27 | If your Centre experienced delays in access to treatment, could you please explain what is the main reason for such delays? (please tick as it applies)   - Pricing Negotiations - The Centre is not authorised to provide the drug - Late referrals to Excellence Centre - Other, please explain |
| 28 | For all those pharmacological treatments unavailable in your facility, could you indicate what is hindering their availability? (multiple choice answer)   - They are not approved by the national medicines agency - They are not included in the national list of reimbursable medicines and/or not reimbursed by national public healthcare payers - They are not reimbursed by local public healthcare payers - They are only reimbursed by private sector healthcare payers - My healthcare facility does not prescribe this treatment - Other, please explain |
| 29 | Are there national legal procedures foreseen for patients to get access to drugs normally unavailable to them? For example through compassionate use, hospital exemption or other schemes? Please explain. |
|  | **GENERAL RECOMMENDATIONS** |
| 30 | Do you have any additional comments to share about the challenges surrounding access to treatment? |
| 31 | In your opinion, what can expedite access to treatment? Please elaborate. |

**Table S2 : Responding centres**

| **Country** | **Number of MetabERN centres** | **Number of responding centres** | **Number of responses** | **Number of complete responses** |
| --- | --- | --- | --- | --- |
| **BE** | 6 | 4 | 4 | 2 |
| **BG** | 1 | 1 | 1 | 1 |
| **CZ** | 1 | 1 | 2 | 1 |
| **DE** | 10 | 8 | 8 | 7 |
| **DK** | 1 | 1 | 1 | 1 |
| **ES** | 5 | 3 | 4 | 4 |
| **FR** | 9 | 6 | 8 | 6 |
| **GB** | 6 | 3 | 5 | 5 |
| **HR** | 1 | 1 | 1 | 1 |
| **HU** | 1 | 1 | 3 | 3 |
| **IT** | 11 | 6 | 7 | 7 |
| **LT** | 1 | 1 | 1 | 1 |
| **NL** | 5 | 5 | 6 | 4 |
| **NO** | 2 | 2 | 2 | 0 |
| **PL** | 1 | 1 | 1 | 0 |
| **PT** | 5 | 5 | 7 | 5 |
| **SE** | 2 | 2 | 2 | 0 |
| **SL** | 1 | 1 | 2 | 2 |
| **Total** | **69** | **52** | **65** | **50** |

**Table S3 : List of the 28 EMA-approved OMPs for HMDs**

| **ACTIVE SUBSTANCE** | **ORPHAN INDICATION** | **PRODUCT NAME COMPANY** |
| --- | --- | --- |
| Carglumic acid | N-acetylglutamate synthase deficiency | Carbaglu  Orphan Europe |
|  | Isovaleric acidaemia |  |
|  | Methylmalonic acidaemia |  |
|  | Propionic acidemia, aciduria, propionyl-CoA carboxylase deficiency |  |
| Chenodeoxycholic acid | Cerebrotendinous xanthomatosis | Chenodeoxycholic acid, Leadiant |
| Cholic acid | Bile acid synthesis deficiencies | Cholbam, Retrophin Inc.  Orphacol, CTRS |
| Glycerol Phenylbutyrate | Argininosuccinic aciduria | Glycerol phenylbutyrate, Ravicti, Horizon Pharma  Pheburane, Lucane Pharma |
|  | Arginimemia |  |
|  | Citrullinaemia type 1 |  |
|  | Citrulinemia type 2, citrin deficiency |  |
|  | Ornithine transcarbamylase deficiency |  |
|  | HHH : Hyperornithinemia-hyperammonemia- homocitrullinuria |  |
|  | Carbamoyl-phosphate-synthase-1 deficiency |  |
| Sodium Phenylbutyrate | Deficiency in enzymes of the urea cycle | Buphenyl, CMIC  Ammonaps, Biovitrum |
| Sapropterin (tetrahydrobiopterin) | Hyperphenylalaninaemia, mild PKU | Sapropterin,  Kuvan, Biomarin |
| Nitisinone | Tyrosinemia type 1 | Orfadin,  Biovitrum |
| Alipogene tiparvovec | Gene therapy  Lipoprotein lipase deficiency | Glybera  Uniqure |
| Cysteamine hydrochloride (mercaptamine hydrocholoride) | Cystinosis, ophtalmic | Cystadrops  Orphan Europe |
| Cysteamine bitartrate (mercaptamine bitartrate) | Nephropathic cystinosis | Cystagon |
| Eliglustat | Substrate reduction therapy Gaucher disease | Cerdelga  Genzyme |
| Migalastat (1-Deoxygalactonojirimycin) | Substrate reduction therapy  Fabry disease | Galafold  Amicus therapeutics |
| Miglustat | Substrate reduction therapy Niemann-Pick disease type C Gaucher disease | Zavesca  Actelion |
| Sebelipase (recombinant human lysosomal acid lipase) | Enzyme replacement therapy  Lysosomal acid lipase deficiency | Kanuma  Actelion |
| Cerliponase alfa (recombinant human tripeptidyl-peptidase 1, rhTPP1) intraventricular | Enzyme replacement therapy  Neuroceroid lipofuscinosis type 2  (CNL2) | Brineura  Biomarin |
| Imiglucerase | Enzyme replacement therapy  Gaucher disease | Cerezyme  Genzyme |
| Velaglucerase Alfa | Enzyme replacement therapy  Gaucher disease | VPRIV  Shire |
| Alglucosidase Alpha | Enzyme replacement therapy  Pompe disease | Myozyme  Genzyme |
| Idursulfase | Enzyme replacement therapy  MPS II (Hunter syndrome) | Elaprase  Shire |
| Elosulfase alfa (Recombinant human N-acetylgalactosamine-6-sulfatase) | Enzyme replacement therapy  MPS IVA (Morquio A syndrome) | Vimizin  Biomarin |
| Galsulfase | Enzyme replacement therapy  MPS VI (Maroteaux-Lamy syndrome) | Naglazyme  Biomarin |
| Laronidase | Enzyme replacement therapy  MPS I (Hurler syndrome) | Aldurazyme  Genzyme |
| Agalsidase alpha | Enzyme replacement therapy  Fabry disease | Replagal  Shire |
| Agalsidase beta |  | Fabrazyme  Genzyme |
| Afamelanotide ([Nle4, D-Phe7] -alpha-melanocyte stimulating hormone) | Erythropoietic protoporphyria | Scenesse  Clinuvel |
| Autologous CD34+ cells transfected with retroviral vector containing adenosine deaminase gene | SCID due to adenosine deaminase (ADA) deficiency | Strimvelis  GSK |
| Idebenone | Leber Hereditary Optic Neuropathy | Mnesis, Raxone  Takeda, Santhera |

**Table S4 : list of MetabERN centres and number of questionnaires completed**

| Country | MetabERN centres | Number of questionnaires completed |
| --- | --- | --- |
| BE | CHU Liège | 2 |
|  | Cliniques universitaires St Luc-Univ. catholique Louvain | 1 |
|  | Ghent University hospital | 0 |
|  | Metabolic Center ULB-VUB | 0 |
|  | University Hospital of Antwerp UZA | 0 |
|  | UZ Leuven | 1 |
| BG | University hospital "Alexandrovska"in Sofia | 1 |
| CZ | General University Hospital in Prague-GUH | 2 |
| DE | Helios Dr. Horst Schmidt Kliniken | 1 |
|  | Charité-Universitätsmedizin Berlin | 1 |
|  | Hannover Medical School | 1 |
|  | Medical Center University of Freiburg | 1 |
|  | Muenster University Hospital | 0 |
|  | Otto-von-Guericke-University Magdeburg | 1 |
|  | Universitätskinderklinik Giessen | 1 |
|  | Universitätsklinikum Heidelberg | 1 |
|  | University Medical Center Hamburg-Eppendorf | 0 |
|  | University Medical Center Mainz | 1 |
| DK | Copenhagen University Hospital, Rigshospitalet | 1 |
| ES | University Clinical Hospital of Santiago de Compostela. | 0 |
|  | Hospital Sant Joan de Déu, Barcelona | 0 |
|  | Hospital Universitario Cruces, Vizcaya | 1 |
|  | Hospital Universitario Vall d'Hebron, Barcelona | 1 |
|  | Hospital Universitario "12 de Octubre" Madrid | 2 |
| FR | Hôpitaux Universitaires Paris Ile de France Ouest | 1 |
|  | Hôpitaux Universitaires Paris Nord Beaujon | 2 |
|  | Hôpitaux Universitaires Lyon | 0 |
|  | Hôpitaux Universitaires Paris Nord Antoine-Béclère | 0 |
|  | Hôpitaux Universitaires Paris Nord Louis-Mourier | 0 |
|  | Hôpitaux Universitaires Paris Necker-Enfants Malades | 2 |
|  | Hôpitaux Universitaires Paris Robert-Debré | 1 |
|  | Hôpitaux Universitaires Lille | 1 |
|  | Hôpitaux Universitaires Marseille | 1 |
| GB | Cambridge University Hospitals | 1 |
|  | Birmingham Children’s Hospital | 3 |
|  | University Hospitals Bristol, Bristol Children's Hospital | 0 |
|  | Central Manchester University Hospitals | 1 |
|  | Great Ormond Street Hospital London | 0 |
| HR | University Hospital Center Zagreb | 1 |
| HU | Hungary-University of Debrecen | 3 |
| IT | Azienda Sanitaria Universitaria Integrata di Udine | 0 |
|  | Meyer Children's Hospital Firenze | 0 |
|  | ASST Monza San Gerardo Hospital | 2 |
|  | Azienda Ospedaliera Universitaria "Federico II", Napoli | 1 |
|  | Azienda Ospedaliera Universitaria Integrata di Verona | 1 |
|  | Ospedale Pediatrico Bambino Gesù, Roma | 0 |
|  | Giannina Gaslini Institute, Genova | 0 |
|  | San Paolo Hospital, ASST Santi Paolo e Carlo, Milano | 0 |
|  | University Hospital of Padova | 1 |
|  | Azienda Ospedaliero-Universitaria Pisana | 1 |
|  | Azienda Ospedaliera Universitaria Senese | 1 |
| LT | Vilnius University Hospital Santariskiu klinikos | 1 |
| NL | Erasmus MC, University Medical Center, Rotterdam | 1 |
|  | Maastricht University Medical Center | 1 |
|  | University Medical Center of Groningen | 2 |
|  | University Medical Center Utrecht (UMCU) | 1 |
|  | Academic Medical Center Amsterdam | 1 |
| NO | Oslo University Hospital | 1 |
|  | Helse Bergen HF, Haukeland University Hospital | 1 |
| PO | Krakow University Hospital | 1 |
| PT | Centro Hospitalar Lisboa Norte, EPE | 1 |
|  | Centro Hospitalar do Porto, EPE (CHP) | 2 |
|  | Centro Hospitalar do Sao Joao, E.P.E. | 2 |
|  | Centro Hospitalar e Universitário de Coimbra, E.P.E. | 1 |
|  | Hospital Senhora da Oliveira – Guimarães | 1 |
| SE | Karolinska University Hospital, Solna | 1 |
|  | Sahlgrenska University Hospital University of Gothenburg | 1 |
| Sl | University Medical Centre Ljubljana | 2 |
|  | **Total number of completed questionnaires** | **65** |
